# Gender Differences in Alexithymia, Emotion Regulation, and Impulsivity in Young Individuals with Mood Disorders

**DOI:** 10.3390/jcm14062030

**Published:** 2025-03-17

**Authors:** Luca Di Benedetto, Mario Pinto, Valentina Ieritano, Francesco Maria Lisci, Laura Monti, Elisa Marconi, Daniela Pia Rosaria Chieffo, Silvia Montanari, Georgios D. Kotzalidis, Gabriele Sani, Delfina Janiri

**Affiliations:** 1 Section of Psychiatry, Department of Neuroscience, Università Cattolica del Sacro Cuore, Largo Francesco Vito 1, 00168 Rome, Italy; luca.dibenedetto91@gmail.com (L.D.B.); valentinaieritano.nutrizione@gmail.com (V.I.); fmlisci@gmail.com (F.M.L.); silvia.montanari@yahoo.com (S.M.); giorgio.kotzalidis@gmail.com (G.D.K.); delfina.janiri@unicatt.it (D.J.); 2 Department of Psychiatry, Fondazione Policlinico Universitario Agostino Gemelli IRCCS, Largo Agostino Gemelli 8, 00168 Rome, Italy; mario.pinto@guest.policlinicogemelli.it; 3 Clinical Psychology Unit, Fondazione Policlinico Universitario Agostino Gemelli IRCCS, 00168 Rome, Italy; laura.monti@fbf-isola.it (L.M.); elisa.marconi@policlinicogemelli.it (E.M.); danielapiarosaria.chieffo@policlinicogemelli.it (D.P.R.C.); 4 Department Women Children and Public Health, Catholic University of Sacred Heart, Largo Agostino Gemelli 8, 00168 Rome, Italy

**Keywords:** mood disorders, adolescents, alexithymia, emotion regulation, impulsivity, gender differences, youth psychopathology, early intervention, psychiatric disorders

## Abstract

**Background/Objectives:** Alexithymia, emotion regulation, and impulsivity are key factors in youths with mood disorders. However, gender differences within these dimensions remain insufficiently studied in this population. This study seeks to explore these dimensions in a sample of adolescents and young adults with mood disorders, aiming to identify gender-specific characteristics with important clinical implications. **Methods:** We assessed 115 outpatients aged 13 to 25 years with a DSM-5 diagnosis of mood disorder. The evaluation included the Toronto Alexithymia Scale (TAS-20), the Difficulties in Emotion Regulation Scale (DERS), and the UPPS-P Impulsive Behavior Scale. The associations with suicidal ideation were tested using two different multivariate models. Results were controlled for age and intelligence measures. **Results:** The first model (Wilks’ Lambda = 0.720, *p* < 0.001) revealed significantly higher scores in women than men for TAS-20 (*p* < 0.001), DERS (*p* < 0.001), and the UPPS-P subscales “Lack of Premeditation” (*p* = 0.004) and “Lack of Perseverance” (*p* = 0.001). Regression analyses confirmed gender as a significant predictor of these variables, also controlling for age and intelligence. Furthermore, intelligence measure influenced Lack of Premeditation and age influenced Lack of Perseverance. **Conclusions:** Women with mood disorders exhibit greater alexithymia, emotional dysregulation, and impulsivity, particularly in difficulties with planning and task persistence. These findings highlight the need for gender-sensitive interventions that address emotional awareness and impulse control to improve clinical outcomes.

## 1. Introduction

Adolescence and young adulthood are crucial developmental stages, often marked by the onset of mood disorders, affecting around 10–20% of young people globally [1]. About 12.8% of 5–19-year-olds have at least one diagnosed mental disorder, with 8.1% suffering from depressive or anxiety disorders [2,3,4]. These rates have risen over the past two decades, with mental health disorders in 5–16-year-olds increasing from 11.4% in 2017 to 16.7% in 2020 [5], and depressive symptoms among adolescents also rising from 24% in 2001–2010 to 37% in 2011–2020 [6]. Gender differences are prominent, with women twice as likely to be diagnosed with major depressive disorder (MDD) as men, starting in adolescence [7,8,9]. Factors such as social, genetic, and physiological influences contribute to these differences [10,11,12,13,14], although further research remains necessary. In addition to the primary symptoms of mood disorders, there are several psychopathological dimensions that contribute to the onset and maintenance of mood symptoms in adolescents [15]. Key among these are alexithymia, emotional dysregulation, and impulsivity.

Alexithymia is a deficit in emotional recognition and regulation, characterized by difficulties in identifying, describing, and expressing emotions. It is a concept first formulated by Peter Sifneos in the early 1970s [16], meaning a lack of words for emotions, which later received neurobiological support [17]. Alexithymia is linked to maladaptive emotional regulation and a worse clinical presentation, especially in depression [18]. Differential gender effects have been reported, along with higher scores for younger people than older ones [19]. Emotional dysregulation refers to the inability to manage the intensity and quality of emotions such as fear, anger, and sadness leading to excitability and mood instability [20,21,22]. Women were reported to score higher on emotion dysregulation [23]. It intersects with impulsivity, another key psychopathological component. This refers to behaviors or tendencies toward engaging in risky actions without careful consideration, often driven by immediate desires or emotions, and without regard for potential consequences, hence frequently resulting in negative long-term outcomes [24,25]. Gender was found to be a moderator of impulsivity, with stronger effects in men [26]. The interplay among these three dimensions is complex and multifaceted, creating a vicious cycle that perpetuates psychological distress and functional impairment. Exploring their roles in the context of adolescent–early adult mood disorders is crucial for early identification and the development of targeted interventions. Although previous studies already identified alterations in these specific psychopathological dimensions in adolescents [27,28], there is a lack of specific studies focusing on gender differences in this specific population. Knowing that alexithymia, emotion dysregulation, and impulsivity are differentially regulated in the two genders in various populations, we sought to identify gender differences in these psychological dimensions in a mood disorder population of young people who sought psychological help in our service. Therefore, this study sought to elucidate the mechanisms linking these constructs with mood disorders with a focus on gender differences. By investigating how these constructs may differently impact boys and men on one hand and girls and women on the other hand, the study seeks to inform targeted strategies for mitigating their effects, ultimately enhancing clinical outcomes for young individuals affected by mood disorders.

## 2. Materials and Methods

### 2.1. Participants

We assessed 115 young outpatients with a DSM-5 mood disorder diagnosis (depressive disorder or bipolar disorder). Patients were enrolled at the Early Intervention for Mood Disorders Unit at Fondazione Policlinico Universitario Agostino Gemelli IRCCS, Rome, Italy. Patients were screened by trained staff for DSM-5 disorders (Fleiss’ *kappa* = 0.85), and clinical diagnoses were confirmed using the Structured Clinical Interview for DSM-5-Research Version [29]. In addition to a diagnosis of mood disorder, inclusion criteria were the following: (i) age range 13 to 25 years, to include teenage years and to encompass BD onset [30]; (ii) illness in its euthymic phase, as per psychometric evaluation (scores on the Hamilton Depression Rating Scale [HAM-D] ≤ 7 and ≤ 12 on the Young Mania Rating Scale [YMRS]); (iii) all were fluent in Italian; and (iv) all had at least five years of school education. Exclusion criteria included: (i) past loss of consciousness due to traumatic head injury; (ii) lifetime history of significant systemic, internal medicine or neurological disease/disorder; (iii) current cognitive impairment; (iv) recent psychotropic medication changes (i.e., past six weeks); (v) current use of stimulant medications; and (vi) a history of psychosis unrelated to the primary mood disorder.

This study adhered to the Principles of Human Rights, as adopted by the 18th General Assembly of the World Medical Association (WMA), Helsinki, Finland, June 1964, and subsequently amended at the 64th General Assembly of the WMA, Fortaleza, Brazil, October 2013. It was approved by the local ethics committee (protocol number: 5025, date of approval: 16 May 2022) and conducted in agreement with the Fondazione Policlinico Universitario Agostino Gemelli Ethics Committee’s guidelines.

All participants and their parents (in case of participant of minor age, i.e., under the age of 18 years), gave their written informed consent to participate in the study after having received a complete explanation of the study procedures and objectives. No financial compensation was provided, emphasizing the voluntary nature of participation.

### 2.2. Psychopathological and Cognitive Assessment

We used the Toronto Alexithymia Scale (TAS-20; [31,32,33]) a 20-item self-report questionnaire widely used in clinical and psychological research for evaluating difficulties in identifying emotions and describing feelings. The scale provides a total score, with higher scores suggesting greater difficulty with identifying, describing, and processing emotions.

To evaluate the ability to regulate emotions, we administered the Difficulties in Emotion Regulation Scale (DERS; [34,35,36]), a 36-item self-rated scale asking the respondents to respond how they relate to their emotions. This scale involves a measurement of emotion regulation based on an integrative conceptualization, dealing not just with the modulation of emotional arousal, but also investigating awareness, understanding, and acceptance of emotions, as well as the ability to act in desired ways, regardless of the subjects current emotional state [36]. A higher score indicates greater difficulties in emotion regulation.

Additionally, we employed the short form of the UPPS-P Impulsive Behavior Scale, a self-assessment tool that provides a detailed and multidimensional view of impulsivity [37,38,39]. The scale is composed of five dimensions that reflect the different ways in which impulsivity can manifest: (1) Negative Urgency: the tendency to act impulsively when experiencing negative emotions, such as anger or sadness; (2) Positive Urgency: the tendency to act impulsively when experiencing very positive emotions, such as excitement or enthusiasm; (3) Lack of Premeditation: the tendency not to consider the consequences of one’s actions before acting; (4) Lack of Perseverance: difficulty in maintaining attention and completing boring or challenging tasks; and (5) Sensation-Seeking: an inclination to seek new and exciting experiences, even if they are risky. Each subscale has its own score, with higher scores in one or more dimensions indicating a greater tendency toward impulsivity in that specific area.

Finally, we used the Raven’s Standard Progressive Matrices (RSPM; [40,41]) to assess the cognitive abilities and logical–abstract reasoning of the participants in our study. It consists of 60 visually presented geometric problems, divided into 5 progressively more difficult sets (A, B, C, D, and E), with 12 items in each set. In sections A and B, each item is presented as a 2 × 2 matrix, requiring the test-taker to identify the missing part of an image from a set of 6 possible answers. Sections C, D, and E involve 3 × 3 matrices, which require the application of increasingly complex abstract reasoning and problem-solving strategies. For these matrices, the missing entry must be selected from 8 possible options. We selected this measure since it is the most consistently used in literature for fluid intelligence and used it as an independent variable to assess prediction of the explored psychological dimensions. Notably, previous research [42] showed a positive relationship between fluid intelligence, as measured by the RSPM, and emotional awareness, suggesting that cognitive abilities play a role in the identification and understanding of emotions [42,43].

### 2.3. Statistical Analyses

First, we conducted a series of one-way ANOVA with gender as the independent variable and age or RSPM scores as the dependent variable to assess the presence of statistically significant differences in mean age or RSPM scores between the two gender groups, with significance set at *p* < 0.05.

Second, to minimize the likelihood of Type I errors, we conducted a multivariate analysis of variance (MANOVA) using all the continuous variables derived from the psychopathological assessment as dependent variables and gender as the independent factor. If the initial model was significant, we conducted a series of one-way ANOVAs to compare the means between the two gender groups. Like all other analyses, the level of significance of MANOVA was set at *p* < 0.05.

Finally, we conducted a series of linear regression analyses to assess the contribution of age, gender, and scores on the RSPM as predictor variables for each of the scales used in the study as outcome variables. For each regression analysis, the score on each scale was used as the dependent variable, while age, gender, and RSPM scores were included as independent variables. This approach allowed us to explore the influence of each predictor on the different constructs assessed through the scales, controlling for the combined effects of age, gender, and logical–abstract reasoning ability. For comparative measurements, we applied a statistical model with a Bonferroni correction (adjusted *p*-value: *p* < 0.05/number of comparisons) to reduce the likelihood of Type I errors. Multivariate normality in the analyses was regularly assessed [44]. Multicollinearity between the predictor variables was assessed [45,46] using Tolerance [47] and VIF (Variance Inflation Factor; [47]) values. Multicollinearity occurs when predictor variables are highly correlated, which can negatively affect the reliability of regression coefficient estimates [45]. Multicollinearity between the predictor variables was assessed using Tolerance and VIF (Variance Inflation Factor) values. Tolerance indicates how independent each predictor variable is from the others [46], while VIF measures the inflation of variance due to collinearity [47]. According to the literature, a Tolerance value below 0.1 and a VIF greater than 5 indicate problematic multicollinearity [45,46]. All statistical analyses were generated using JASP (Version 0.19.1; JASP Team, Liverpool, UK, 2024) and SPSS (Version 29.0.1.0; IBM Corp, Armonk, NY, USA, 2023).

## 3. Results

In the total sample of participants the mean age was 16.79 (SD = 2.29) and the mean on RSPM was 75.24 (SD = 22.33), while 76 participants were girls or women (66%) and 39 boys or men (34%). The one-way ANOVA Gender × Age was not significant—F_(1, 113)_ = 3.059, *p* = 0.086, η^2^_p_ = 0.026; Test for Equality of Variances (Levene’s) *p* = 0.984] indicating no significant difference in age between female participants (F = 16.53, SD = 2.24) and male participants (M = 17.31, SD = 2.32). Similarly, the one-way ANOVA Gender × RSPM—F_(1, 113)_ = 3.091, *p* = 0.096, η^2^_p_ = 0.029; Test for Equality of Variances (Levene’s) *p* = 0.204—indicated no significant difference on RSPM scores between female participants (M = 76.28, SD = 22.57) and male participants (M = 81.03, SD = 20.96). The large majority in our sample were diagnosed with MDD (N = 79; 68.70%), while 7 (6.09%) received diagnosis of bipolar I and 29 (25.28%) of bipolar II disorder.

Preliminary MANOVA revealed a significant global gender effect (Wilks’ Lambda = 0.720, F = 5.948, df = 7, *p* < 0.001). Based on this result, we subsequently conducted a series of one-way ANOVAs to compare means among groups (Table 1).

**Figure 1:**
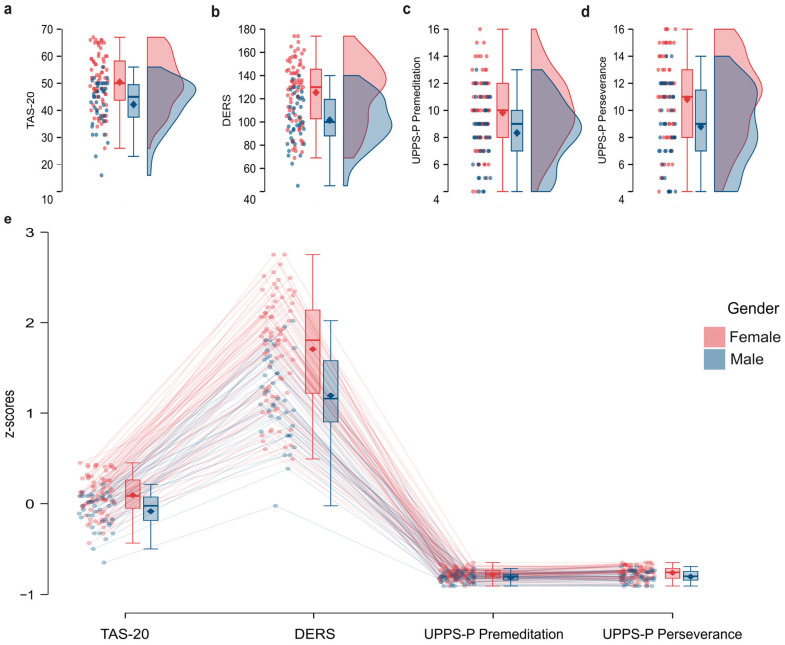
Raincloud plots showing the distribution of scores obtained by females (F) and males (M): (**a**) on the Toronto Alexithymia Scale (TAS-20), (**b**) on the Difficulties in Emotion Regulation Scale (DERS), (**c**) on the Lack of Premeditation dimension of the UPPS-P Impulsive Behavior Scale (UPPS-P Premeditation), and (**d**) on the Lack of Perseverance dimension of the UPPS-P Impulsive Behavior Scale (UPPS-P Perseverance). (**e**) Distributions of aggregated z-scores, computed across the TAS-20, DERS, and UPPS-P subscales, are shown separately for each scale and for both groups (women and men), with corresponding box plots.

The series of one-way ANOVAs highlighted the presence of a significant difference between females and males in the scores obtained on the TAS-20 (*p* < 0.001; Figure 1a), on the DERS (*p* < 0.001; Figure 1b), in UPPS-P—Lack of Premeditation (*p* = 0.004; Figure 1c), and in UPPS-P—Lack of Perseverance (*p* = 0.001; Figure 1d). Females obtained significantly higher scores compared to males. No significant difference was present for Negative Urgency, Positive Urgency or Sensation-Seeking subscales of UPPS-P (all *p* > 0.007; adjusted *p*-value: *p* < 0.05/number of comparisons; Table 1).

The linear regression analysis conducted on the TAS-20 scores obtained by participants to examine the contribution of Age, Gender, and RSPM scores revealed a significant regression model—(F_(3, 114)_ = 7.365, *p* < 0.001)which explained a significant portion of the variance in the scores obtained (R^2^ = 0.166, Adjusted R^2^ = 0.143). Specifically, Gender was found to be a significant predictor (B = −7.608, *t* = 3.933, *p* < 0.001), showing that women scored higher (M = 50.447, SD = 9.991) than men (M = 42.154, SD = 8.768; Figure 1a). Conversely, Age (β = −0.043, *t* = −0.483, *p* = 0.630) and RSPM scores (β = −0.132, *t* = −1.469, *p* = 0.145) showed no significant association with TAS-20 scores.

The linear regression analysis performed on the DERS scores obtained by our participants also revealed a significant regression model—(F_(3, 114)_ = 7.351, *p* < 0.001)—which explained a significant portion of the variance (R^2^ = 0.166, Adjusted R^2^ = 0.143). Gender was the only significant predictor (B = −23.479, *t* = −4.491, *p* < 0.001), showing that women obtained higher scores (M = 125.395, SD = 27.449) as compared with men (M = 101.590, SD = 21.666; Figure 1b). No significant relationship was found with Age (β = −0.040, *t* = −0.453, *p* = 0.652) and RSPM scores (β = 0.005, t = 0.061, *p* = 0.952).

For the Negative Urgency dimension of the UPPS-P, no significant regression model was found—F_(3, 114)_ = 3.711, *p* = 0.014—given that the corrected *p*-value threshold for significance was *p* = 0.007. Similarly, no significant regression model was identified for the Positive Urgency dimension of the UPPS-P—F_(3, 114)_ = 2.353, *p* = 0.076. Furthermore, we found a significant regression model for the scores obtained in the UPPS-P Lack of Premeditation dimension—F_(3, 114)_ = 5.302, *p* = 0.002—which explained a significant portion of the variance (R^2^ = 0.125, Adjusted R^2^ = 0.102). Gender also emerged as a significant predictor (B = −2.08, t = −2.366, *p* = 0.020), demonstrating that women obtained higher scores (M = 9.829, SD = 2.650) compared to men (M = 8.333, SD = 2.432; Figure 1c). Additionally, a significant inverse relationship was found with the RSPM scores (β = −0.213, t = −2.320, *p* = 0.022), suggesting higher scores on the Lack of Premeditation dimension of the UPPS-P for participants with lower RSPM scores and vice versa. No significant association was observed with Age (β = −0.072, t = −0.790, *p* = 0.431).

A significant regression model was found for the Lack of Perseverance dimension of the UPPS-P—F_(3, 114)_ = 5.715, *p* < 0.001—explaining a substantial portion of the variance (R^2^ = 0.134, Adjusted R^2^ = 0.110). Specifically, gender was identified as a significant predictor (B = −2.076, t = −3.393, *p* < 0.001), indicating that women scored higher (M = 10.816, SD = 3.131) than men (M = 8.795, SD = 2.957; Figure 1d). Age was also found to be a significant direct predictor (β = 0.195, t = 2.144, *p* = 0.034), highlighting that lower scores for Lack of Perseverance are associated with younger age, and vice versa. However no significant relationship was observed with RSPM scores (β = −0.126, t = −1.385, *p* = 0.169). Lastly, no significant regression model was identified for the Sensation Seeking dimension of the UPPS-P—F_(3, 114)_ = 1.539, *p* = 0.208.

The assessment of multivariate normality indicated no significant deviation from normality in the data distribution, as evaluated using the Shapiro–Wilk test for multivariate variables [48]. The test yielded a Shapiro–Wilk statistic of W = 0.987 with a *p*-value of 0.304.

The multicollinearity analysis showed that the obtained values in this study (Gender—Tolerance = 0.949, VIF = 1.054; Age—Tolerance = 0.943, VIF = 1.060; RSPM—Tolerance = 0.935, VIF = 1.069) were all above the 0.1 threshold for Tolerance, indicating low multicollinearity, and the VIF values for the independent variables were below 4, suggesting no significant collinearity issues.

## 4. Discussion

The present research aimed to investigate the psychopathological components of alexithymia, emotional dysregulation, and impulsivity in a sample of young outpatients diagnosed with mood disorders specifically highlighting how these components differ across genders. The study thus explored the relationships between these variables and aimed to provide a deeper understanding of the cognitive and emotional mechanisms that contribute to mood disorders in adolescents, considering gender differences in both the expression of mood disorders and the underlying cognitive and emotional processes. Our results, obtained through assessment scales for the three psychopathological components, demonstrate significant gender differences. Overall, women showed higher scores than men in alexithymia, emotion dysregulation, and on the UPPS-P subscales “Lack of Premeditation” and “Lack of Perseverance”. These findings suggest that gender is a predictor of these dimensions, with women experiencing greater emotional and cognitive challenges related to mood disorders, particularly MDD.

Analyzing specifically the different psychopathological dimensions, starting with alexithymia, our linear regression analysis of the TAS-20 scores revealed that gender was a significant predictor of alexithymia, with women showing higher scores compared to men. This finding aligns with previous research indicating that healthy girls often experience greater difficulty identifying and describing their emotions [49,50]. However, our study specifically focuses on adolescents with mood disorders, which may present a different emotional processing profile compared to the general adolescent population. In older populations with MDD, the man vs. woman in alexithymia scores was much smoothened [51]. While our findings are consistent with earlier studies, they contrast with other research suggesting that adult men exhibit higher levels of alexithymia than women [52]. Specifically, it has been shown that men tend to score higher on externally oriented thinking, a core component of alexithymia, compared to women [53,54]. These conflicting findings underscore the complexity of gender differences in emotional processing and suggest that the relationship between alexithymia and gender may vary depending on factors, such as population characteristics, measurement methods, diagnosis, and age.

Similarly, the scores obtained on the DERS scale for emotional dysregulation, revealed that gender was the only significant predictor of difficulties in emotional regulation, with young girls again scoring higher than men. Given that our sample consists of individuals affected by mood disorders, this may contribute to an amplified gender difference in emotional dysregulation for several reasons. Research has shown that women are more predisposed to developing mood disorders compared to men, which increases their vulnerability to difficulties in emotional regulation [15,55]. Moreover, they generally exhibit higher levels of emotional dysregulation compared to men [56,57], a trend that is particularly evident in our sample, where women with mood disorders show greater emotional dysregulation. This combination of heightened susceptibility to mood disorders and the inherent tendency for emotional dysregulation in women likely explains the more pronounced gender differences in emotional regulation observed in our study. These findings suggest that gender-related factors play a crucial role in understanding emotional dysregulation, particularly in adolescents with mood disorders.

The absence of a significant relationship with age or cognitive ability (RSPM scores) further emphasizes the strong role of gender in emotional regulation difficulties within this population, suggesting that women patients may face particular challenges in managing their emotional responses, potentially contributing to mood instability.

Regarding impulsivity, literature tends to support that men, both in adolescence [58] and adulthood [59], generally behave more impulsively than women. In particular, previous studies highlighted a preponderance in Positive Urgency and Sensation Seeking components in young [60] and adult men [61]. Conversely, when examining the Negative and Positive Urgency dimensions, our study revealed a non-significant effect of gender on these impulsive behaviors as strong. This might imply that impulsivity triggered by emotional urgency (whether positive or negative) could be less gender-specific in adolescents and young adults with mood disorders. Conversely, we found significant gender differences in the Lack of Premeditation and Lack of Perseverance dimensions. In both cases, women exhibited higher scores, indicating that they may be more prone to impulsive actions without considering the consequences (i.e., Lack of Premeditation) and may exhibit a greater tendency to disengage from tasks or goals more swiftly (i.e., Lack of Perseverance) compared to men. The tendency for women to engage in particular in Lack of Premeditation and Lack of Perseverance may be related to difficulties in managing emotional responses, as impulsivity in these domains often arises from emotional dysregulation [62,63,64,65]. Additionally, RSPM scores were found to significantly predict the Lack of Premeditation dimension, with participants scoring lower on RSPM (indicating lower cognitive ability) showing higher impulsivity in this dimension. This suggests that cognitive functioning may influence the ability to plan ahead and resist immediate urges, further highlighting the complex interplay between cognitive capacity, impulsivity, and emotional regulation [66].

Age also emerged as a significant predictor of Lack of Perseverance, with older participants displaying higher scores, suggesting that age may play a role in how individuals with mood disorders engage with tasks and persist in the face of challenges. Lack of perseverance has been linked to younger age in a college sample [67], but with advancing age people adaptively tend to persevere more [68,69]. The increase in score observed among older individuals in this study may be attributed to the negative impact of the illness, as those with longer duration of the underlying mood disorder may experience greater difficulties in maintaining perseverance.

While our findings align with existing literature, differences with adult literature versus adolescence and early adulthood have also emerged. In particular, alexithymia, perseverance, impulsivity, and emotion regulation tend to be differently affected by age in the two genders. These findings have significant implications for the clinical treatment of individuals with mood disorders. Since women demonstrate higher levels of alexithymia, impulsivity, and difficulties in emotional regulation, tailored therapeutic interventions should be developed to address these gender-specific challenges.

While this study provides valuable insights, several limitations should be acknowledged. First, the cross-sectional design limits causal inferences about the relationships between gender, alexithymia, impulsivity, and emotional regulation. Second, the relatively small sample size from a single site prevents us from generalizing our results to the entire Italian youth population. This might have exposed us to sampling bias. Longitudinal studies could provide a more comprehensive understanding of how these traits interact over time. Additionally, the results may be influenced by self-assessment biases. Although these self-report tools are considered the gold standard in the field, being among the most widely used and validated for assessing psychopathological components, they remain vulnerable to factors, like social desirability or difficulties in accurately reporting emotions and behaviors. We should add that the self- versus clinician-rating issue has not been sufficiently resolved in psychometric research [70,71], and that there are no validated clinician-rating scales investigating alexithymia, impulsivity, and emotion dysregulation.

The composition of the sample, which is predominantly women, represents a limitation. This is consistent with the literature indicating that women are more frequently affected by mood disorders [66,72]. Consequently, caution is required when generalizing these findings to more gender-balanced populations. Future research should examine these relationships in more diverse samples, including varying gender groups, age ranges, and clinical populations, to assess whether these gender differences persist in other contexts.

## 5. Conclusions

In conclusion, our study highlights significant gender differences in alexithymia, impulsivity, and difficulties in emotional regulation in young individuals with mood disorders. Women exhibited higher scores across these dimensions. These findings contribute to the growing body of literature on the psychological profiles of individuals with bipolar disorder, offering insights into potential targets for treatment and intervention. Further research is needed to explore the underlying mechanisms driving these differences and to refine therapeutic approaches.

## Figures and Tables

**Table 1:** Sociodemographic, cognitive and psychopathological characteristics of the total sample of participants.

Variables	Women (N = 76)	Men (N = 39)	F	df	*p* Value	Effect Size (η^2^_p_)
Age, y—mean ± SD	16.53 ± 2.24	17.31 ± 2.32	3.059	1	0.086	0.026
RSPM	76.28 ± 22.57	81.03 ± 20.96	3.091	1	0.096	0.029
TAS-20	50.45 ± 9.99	42.15 ± 8.77	19.248	1	**<0.001**	0.146
DERS	125.39 ± 27.45	101.59 ± 21.66	22.199	1	**<0.001**	0.164
UPPS-P—Negative Urgency	11.33 ± 3.06	10.31 ± 2.44	3.262	1	0.074	0.028
UPPS-P—Positive Urgency	10.51 ± 3.52	9.56 ± 1.93	2.455	1	0.120	0.021
UPPS-P—Lack of Premeditation	9.83 ± 2.65	8.33 ± 2.43	8.670	1	**0.004**	0.071
UPPS-P—Lack of Perseverance	10.82 ± 3.13	8.79 ± 2.96	11.140	1	**0.001**	0.090
UPPS-P—Sensation-Seeking	9.91 ± 3.34	11.02 ± 2.25	3.535	1	0.063	0.030

All significant values in **bold** characters. Abbreviations: DERS, Difficulties in Emotion Regulation Scale; df, degrees of freedom; N, number of observations; RSPM, Raven’s Standard Progressive Matrices; SD, standard deviation; TAS-20, Toronto Alexithymia Scale; UPPS-P, Impulsive Behavior Scale; y, years.

## Data Availability

The data presented in this study are available on request from the corresponding author due to ethical reasons.

## Abbreviations

The following abbreviations are used in this manuscript:

TAS-20	Toronto Alexithymia Scale
DERS	Difficulties in Emotion Regulation Scale
UPPS-P	Impulsive Behavior Scale
MDD	Major Depressive Disorder
HAM-D	Hamilton Depression Rating Scale
WMA	World Medical Association
RSPM	Raven’s Standard Progressive Matrices
VIF	Variance Inflation Factor
